# Interpretable Acoustic-Emission Leak Detection and ED+MLE-Based Regional Localization in Thin Aluminum-Alloy Plates Under Vacuum Pressure-Difference Excitation

**DOI:** 10.3390/s26175458

**Published:** 2026-08-28

**Authors:** Wei Sun, Jian Zhang, Tao Zhang, Xuyan Hou

**Affiliations:** 1 School of Mechatronics Engineering, Harbin Institute of Technology, Harbin 150001, China; 2 Beijing Institute of Spacecraft Environment Engineering, Beijing 100094, China; 3 School of Mechanical and Electrical Engineering, Northeast Forestry University, Harbin 150040, China; freezeman007@nefu.edu.cn (J.Z.); zt00663@nefu.edu.cn (T.Z.)

**Keywords:** acoustic emission, leak monitoring, thin-walled aluminum-alloy plate, spacecraft sealed structure

## Abstract

Continuous gas leakage in thin-walled sealed spacecraft structures produces sustained broadband acoustic-emission (AE) signals from which reliable first-arrival picking is difficult. This study presents an interpretable two-stage workflow that decouples leak screening from regional localization. Experiments used a 500 × 500 × 2.5 mm 3A21 aluminum-alloy plate with eight piezoelectric AE sensors under 0.1 MPa pressure difference. Intact, circular-hole, and slit-leak conditions were each tested three times with a 0.5–1.5 s steady-state window. Leak screening employed the 20–200 kHz mean spectral amplitude (Hann window). A provisional threshold of 0.85, set from three intact records, separated all leak records from intact controls. Regional localization used relative logarithmic RMS amplitudes in an energy-decay plus maximum-likelihood-estimation (ED+MLE) cost function on a 10 mm grid. The equivalent attenuation coefficient, empirically selected as 2.3 m^−1^ using the 1 mm central-hole calibration case, gave a 10 mm grid error for that calibration demonstration. With this coefficient fixed, the four transfer conditions yielded mean localization errors of 19.6–45.7 mm. The results provide preliminary feasibility evidence for leak detection and regional localization under the controlled laboratory conditions investigated in this study. Further validation requires independent channel calibration, larger background datasets, leak-rate measurements, parameter-sensitivity analysis, and testing on more representative structures.

## 1. Introduction

Micro-holes, slit-like defects, and micro-cracks can arise in spacecraft sealed cabins, tanks, pipelines, and local thin-walled panels due to micrometeoroid/debris impacts, thermal-cycling fatigue, manufacturing flaws, or stress concentrations near joints. When a pressure difference exists across the wall, such defects may drive continuous gas leakage, potentially compromising cabin-pressure retention, propellant-storage safety, and overall mission reliability [1,2,3,4]. Unlike ground-based facilities, in-orbit spacecraft structures cannot readily be inspected, disassembled, or re-instrumented. Any practical monitoring method must therefore deliver, with a limited number of sensors and modest computational resources, three essential outputs: whether a leak is present, how strong the leak response is, and the approximate region in which the source lies [1,2].

Acoustic emission (AE) sensing is attractive for this task because it is passive, sensitive to local dynamic events, compatible with surface-mounted piezoelectric transducers, and well-suited to online structural health monitoring [5,6,7,8,9,10,11]. Many localization studies [7,8,9,10,11,12,13,14,15,16,17,18] treat the leak or defect as an acoustic source whose position is estimated from time-difference-of-arrival (TDOA), energy/amplitude decay, beamforming, or array-signal-processing features. TDOA methods work extremely well when the signal contains a clear, impulsive onset, as in impact or burst-type AE events [8,9,14,16,17,18]. In thin plates, however, continuous leak AE signals typically appear as broadband, random-like responses influenced by Lamb-wave dispersion, multi-modal coupling, boundary reflections, and channel-to-channel coupling variability [2,3,6,13,15,19]. These effects render first-arrival picking unreliable and considerably degrade the accuracy of purely TDOA-based localization in small thin-plate leak scenarios.

Energy-decay methods offer a complementary route, inferring the source region from the spatial distribution of received amplitudes rather than from arrival times [7,20,21]. They are computationally light and physically interpretable—attributes that are highly advantageous for embedded spacecraft health-monitoring systems. To date, however, amplitude- or energy-based leak-localization studies have largely focused on free-field gas leaks, pipelines, or transient AE sources. Their propagation media, sensor-coupling conditions, attenuation behavior, and boundary conditions differ substantially from those encountered in piezoelectric AE monitoring on a bounded thin plate under vacuum pressure-difference excitation [2,3,5,6,13,22,23,24]. The specific methodological gap addressed here is the adaptation of a relative-amplitude ED+MLE localization framework to continuous leak AE signals in a thin aluminum plate, while keeping the workflow simple enough for practical engineering deployment.

A second practical issue concerns the interpretation of leak severity and defect geometry. Hole- and slit-type leaks may originate from different damage mechanisms, but any single mean-amplitude feature is influenced by aperture area, pressure difference, source-sensor distance, propagation direction, and coupling conditions [7,20,24,25]. In this study, interpretability refers to the explicit physical traceability between the measured acoustic feature, the propagation model, and the resulting localization estimate. Specifically, the screening feature is an explicitly defined band-averaged spectral quantity associated with the measured broadband leak response, while the ED+MLE model explicitly represents spatial amplitude decay. Consequently, deviations in the localization result can be discussed in terms of measurable factors such as channel sensitivity, coupling condition, equivalent attenuation, and sensor-array geometry. This definition describes the physical transparency of the present workflow rather than implying that other localization approaches are inherently non-interpretable. Accordingly, the band-averaged spectral amplitude is used as a leak-state and response-intensity indicator, not as a stand-alone hole/slit classifier.

On this basis, the present study proposes and evaluates a two-stage workflow for thin-plate leak monitoring. A 3A21 aluminum-alloy plate serves as an idealized ground-equivalent local panel, and AE responses are acquired from intact, circular-hole, and slit-leak conditions under an atmospheric-pressure-to-vacuum pressure difference. A low-dimensional frequency-domain feature is then employed to separate intact and leak states. Finally, an ED+MLE regional localization model is constructed from relative RMS amplitudes so that continuous leak signals can be localized without first-arrival picking. The main contributions are: (1) construction of an eight-channel piezoelectric AE leak dataset for an aluminum thin plate under vacuum pressure-difference excitation; (2) demonstration that the 20–200 kHz band-averaged spectral amplitude supplies an interpretable leak/no-leak and response-intensity indicator in this controlled dataset; and (3) adaptation of relative-amplitude ED+MLE regional localization to continuous thin-plate leak AE signals, with explicit consideration of aperture, source position, channel inconsistency, and attenuation-parameter limitations.

## 2. Materials and Methods

### 2.1. Ground-Equivalent Experimental Design for Thin-Walled Sealed-Structure Leaks

A 3A21 aluminum-alloy thin plate was chosen as an idealized local panel rather than a full spacecraft structure. This choice provides controllable boundary conditions, sensor coordinates, and defect locations, making it suitable for evaluating the proposed signal-processing and localization framework. At the material and local-structure level, the plate represents a simplified metallic thin-walled sealed component, such as a cabin-wall panel, tank panel, or local sealed plate section. At the driving-mechanism level, the vacuum pump establishes a pressure difference across the plate, corresponding to gas leakage from a pressurized cavity toward a lower-pressure environment [2,3].

The tests simulated leakage from the atmospheric-pressure side to the vacuum side under an approximately 0.1 MPa pressure difference. This condition generated stable leak excitation and enabled comparisons among different aperture and slit geometries under the same nominal driving force. This study evaluates the feasibility of the proposed method on a flat aluminum-alloy plate under a steady pressure difference; structural complexities, environmental effects, and multiple simultaneous leak sources were outside the present scope. Leak-rate calibration and pressure–time records were not available in the present dataset; consequently, the results do not support quantitative leak-severity assessment.

### 2.2. Specimen and Defect Design

The specimen measured 500 mm × 500 mm × 2.5 mm. A plate coordinate system was established with one corner of the specimen taken as the origin; all defect and sensor coordinates are reported in millimeters. Four circular hole-type defects and one slit-type defect were prepared, with an intact condition retained as the control. The defect set comprised a 1.0 mm central hole at (250, 250) mm, a 0.3 mm upper-left eccentric hole at (100, 350) mm, a 0.5 mm right-side eccentric hole at (400, 250) mm, a 0.1 mm × 5 mm lower slit at (250, 100) mm, and a 0.1 mm lower-left micro-hole at (100, 100) mm. This configuration was used to examine the effects of aperture, geometry, and source position on the spectral response and ED+MLE localization.

**Figure 1:**
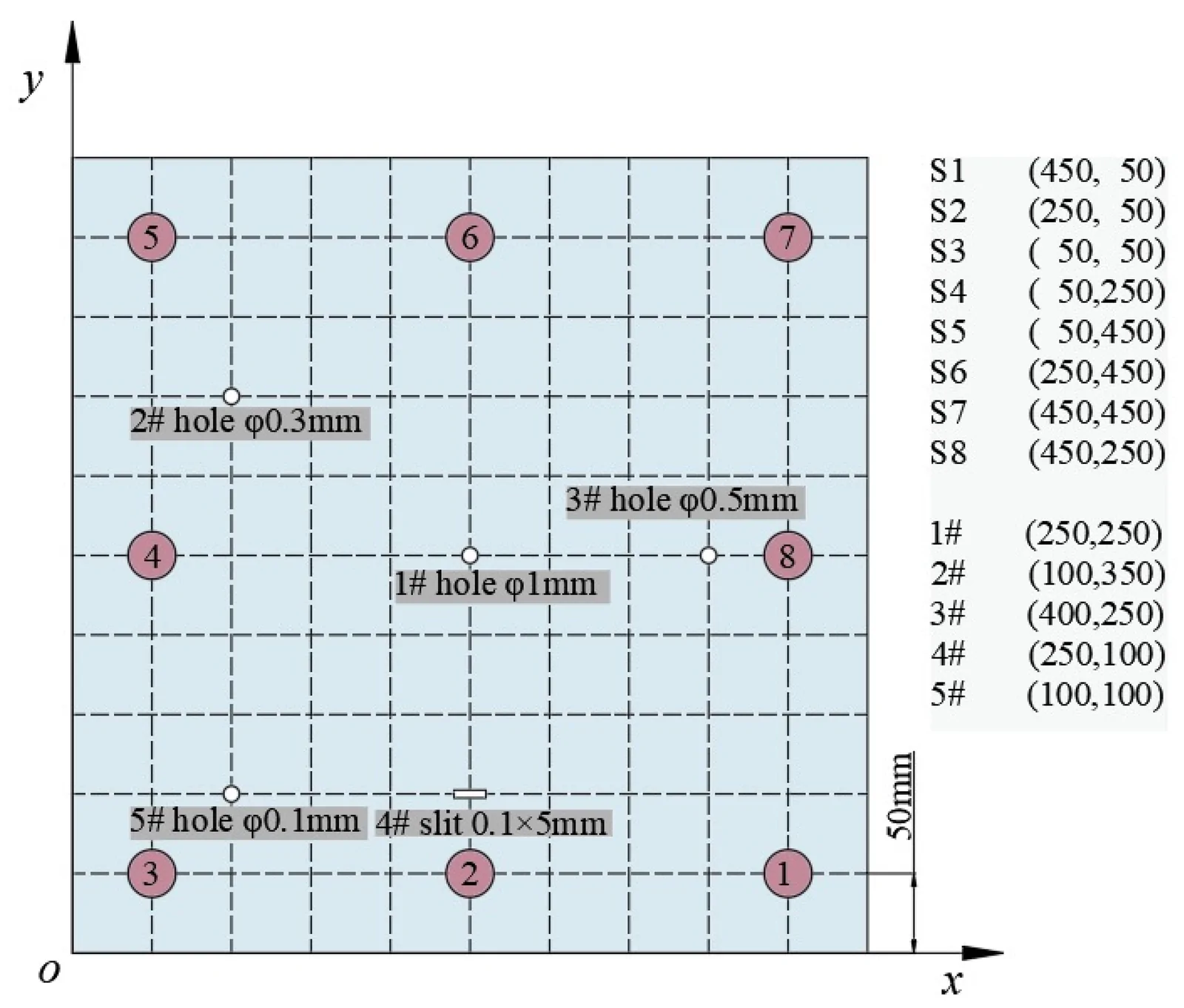
Integrated defect and sensor coordinate layout for the 500 mm × 500 mm aluminum-alloy plate. The red numbered circles denote AE sensors S1–S8, while the open circles indicate the defect locations.

Figure 1 shows the integrated defect-sensor coordinate layout used for localization. To maintain sufficient geometric constraints for sources distributed across the plate interior, the eight sensors were arranged in a boundary-surrounding pattern [5,6,9]. The sensor coordinates were S1 (450, 50), S2 (250, 50), S3 (50, 50), S4 (50, 250), S5 (50, 450), S6 (250, 450), S7 (450, 450), and S8 (450, 250) mm.

### 2.3. Acoustic Emission Acquisition System and Sensor Array

**Figure 2:**
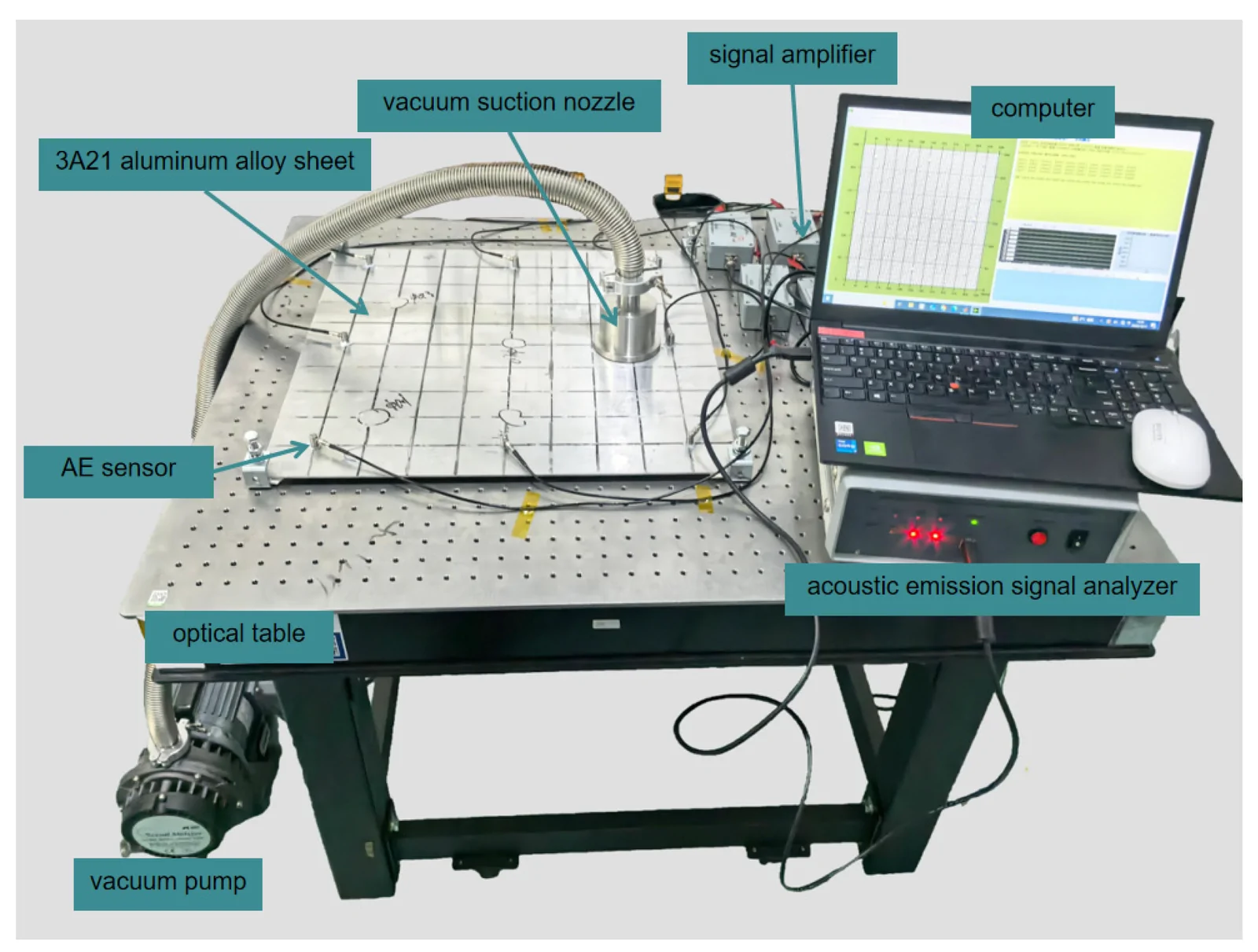
Photograph of the experimental AE leak-testing setup under atmospheric-pressure-to-vacuum pressure-difference excitation.

The AE acquisition system comprised eight RS-54A piezoelectric AE sensors, Smart AE SGM01A-40 dB preamplifiers, and the built-in synchronous full-waveform acquisition module of a DS-series AE analyzer (Beijing Soft Island Times Technology Co., Ltd., Beijing, China). The RS-54A sensors are broadband piezoelectric transducers with a nominal frequency range of 100–900 kHz, and the preamplifiers provide 40 dB gain over a nominal 20–1500 kHz bandwidth. All eight channels were sampled synchronously at 3 MHz with 16-bit A/D conversion and a ±10 V input range; each record lasted at least 1.5 s. The sensors were coupled to the plate surface using an AE couplant, and the laboratory setup is shown in Figure 2. The exact DS-series host submodel and commercial couplant type were not recorded. Because independent channel calibration was not performed, channel gain and coupling inconsistency are considered key uncertainty sources for amplitude-based localization.

The 20–200 kHz analysis band was selected empirically from the measured spectra obtained under the present experimental configuration. This processing-band selection should not be interpreted as an independent calibration of the RS-54A sensor response over the entire 20–200 kHz range. In particular, the response below the nominal lower-frequency limit was not independently characterized in this study, and this is treated as a limitation of the present experiment.

### 2.4. Dataset Description

Three repeated acquisitions were obtained for each condition, and all records were longer than 1.5 s. Because only three repetitions were collected for each condition, the present dataset supports controlled feasibility analysis and preliminary performance assessment rather than statistically mature validation of the detection threshold or localization accuracy.

## 3. Signal Processing and ED+MLE Regional Localization

### 3.1. Two-Stage Monitoring Framework

**Figure 3:**
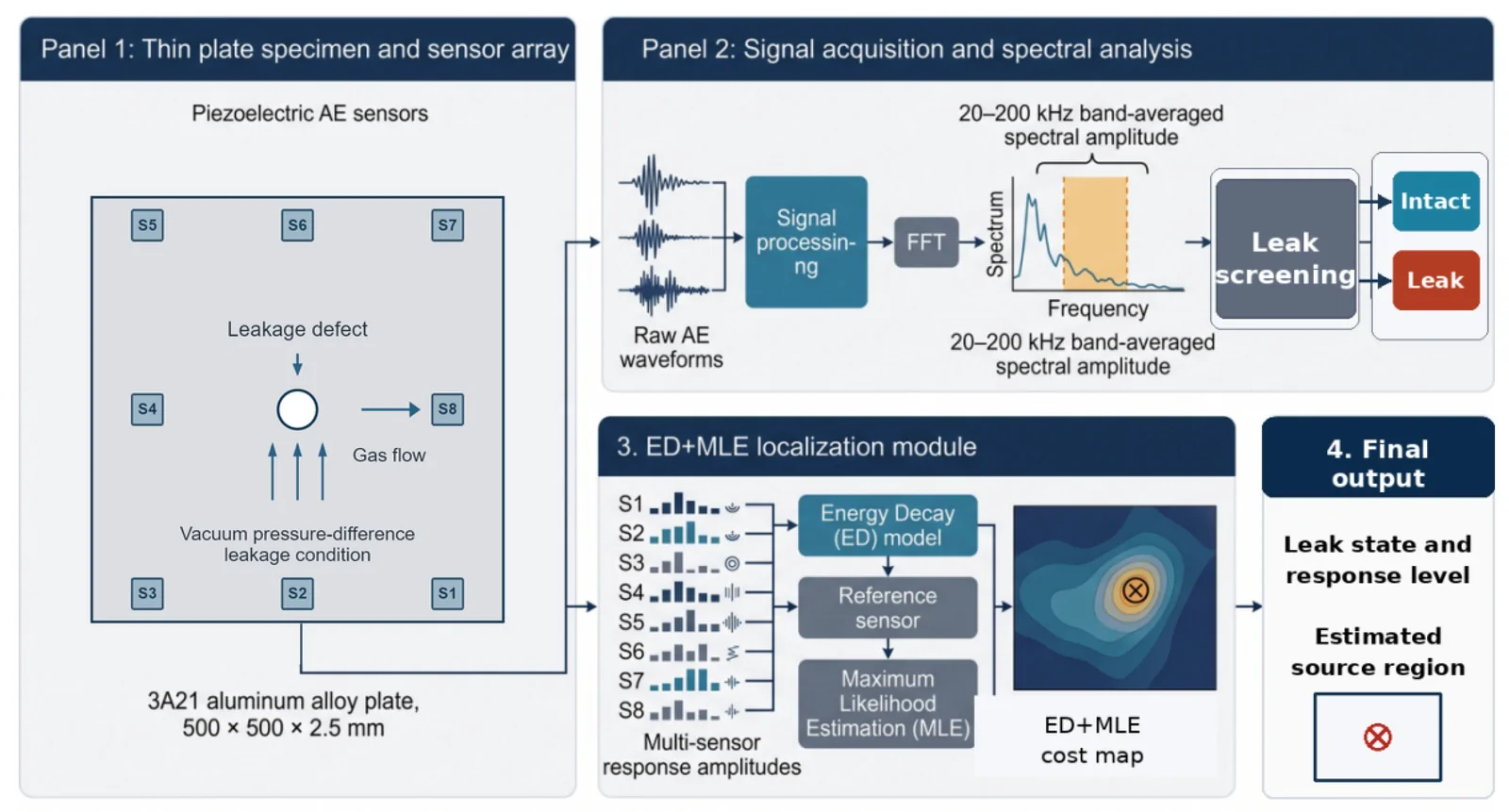
Two-stage workflow for AE-based leak identification and ED+MLE regional localization.

The workflow separates leak screening from source localization (Figure 3). First, a steady-state segment is extracted from the eight-channel AE record, and 20–200 kHz frequency-domain features are used to determine whether a leak is present and to characterize response intensity. Second, for leak-positive records, channel RMS amplitudes from that same segment are converted into relative logarithmic amplitudes and fitted with an ED+MLE spatial cost function over a two-dimensional grid. This separation assigns distinct roles to the two features: the spectral feature identifies leak presence, whereas the RMS amplitude distribution estimates the leak-source region.

### 3.2. Signal Preprocessing and Frequency-Domain Feature Extraction

For each eight-channel AE record, the steady-state portion of the leakage process was extracted and the mean value of each channel was removed to suppress DC bias. A unified 1 s segment from 0.5 s to 1.5 s was selected for all conditions. This window avoids the initial transient disturbance caused by vacuum pump startup and provides an identical signal length across samples. At a sampling rate of 3 MHz, each analysis window contains 3,000,000 points. Let x_i_[n] denote the discrete signal acquired by the i-th sensor. Its frequency-domain representation is

(1) $$X_{i}\left(f_{k}\right)=F\{x_{i}\left[n\right]\}$$

Spectral analysis was performed using a Hann window. The FFT length NFFT was set equal to the 3,000,000 samples in the steady-state window, and a coherent-gain-corrected, normalized single-sided amplitude spectrum was used. The 20–200 kHz band was selected primarily on the basis of the measured spectra in the present experiments rather than from a dedicated Lamb-wave dispersion or plate-resonance analysis. To examine the 200 kHz upper cutoff, the spectral characteristics in the 20–200 kHz and 200–400 kHz ranges were compared using the same records. The leakage-to-baseline ratios for the circular-hole and slit-leak groups decreased from 35.2 and 37.7 dB in the 20–200 kHz band to 13.6 and 17.2 dB in the 200–400 kHz band, respectively. Inter-channel variability did not improve consistently at higher frequencies: the mean CV changed from 0.292 to 0.285 for circular-hole leaks and from 0.376 to 0.511 for the slit-leak condition. Thus, the higher band provides substantially weaker leak-to-baseline contrast and no consistent reduction in channel variability under the present configuration. This supports exclusion of the higher range from the primary feature-extraction band. The selected band is configuration-dependent and should not be transferred directly to other structures or sensor configurations.

(2) $$\bar{A_{i}}=\frac{1}{N_{B}}\sum_{f_{k}\in B}\left|X_{i}\left(f_{k}\right)\right|$$

where B denotes the 20–200 kHz band and NB denotes the number of frequency bins in this band. The array-level mean spectral amplitude is defined as

(3) $$\bar{A_{array}}=\frac{1}{M}\sum_{i=1}^{M}\;\bar{A_{i}}$$

where M = 8 is the number of sensors. To assist the analysis of spectral-shape differences, the peak frequency and peak amplitude were also extracted:

(4) $$f_{p}=\arg\underset{f_{k}\in B}{\max}\left|X_{i}\left(f_{k}\right)\right|,{\;A}_{p}=\underset{f_{k}\in B}{\max}\left|X_{i}\left(f_{k}\right)\right|$$

The array-level feature Āarray is used exclusively for leak/no-leak discrimination and response-intensity characterization. It is not treated as an intrinsic descriptor of defect geometry, because it is also affected by aperture, pressure difference, source-sensor distance, propagation direction, channel gain, and coupling state. All spectral amplitudes are normalized processing features derived from preamplified sensor voltages, not from absolute acoustic source strengths. The same 0.5–1.5 s steady-state segment was used for both spectral screening and RMS-based localization.

### 3.3. Leak-State Discrimination Using Background Statistics

Given the small dataset, a complex supervised classifier was not constructed. Instead, a background-statistics threshold was adopted because it is transparent, easy to implement, and appropriate for preliminary engineering validation. Let Ā_0,j_ denote the array-level mean spectral amplitude of the j-th intact sample and N_0_ be the number of intact samples. The background mean and sample standard deviation are

(5) $$\mu_{0}=\frac{1}{N_{0}}\sum_{j}\;\bar{A_{0,j}},\sigma_{0}={\left[\frac{1}{N_{0}-1}\sum_{j}{\left(\bar{A_{0,j}}-\mu_{0}\right)}^{2}\right]}^{\frac{1}{2}}$$

The leak-detection threshold is set as

(6) $$T=\mu_{0}+k\sigma_{0}$$

where k is the threshold coefficient. A value of k = 3 was used to provide a conservative separation from the observed intact background. In the present dataset, the three intact samples had array-level mean spectral amplitudes of 0.71, 0.78, and 0.75 in the 20–200 kHz band. The mean was μ_0_ = 0.747 and the sample standard deviation was σ_0_ = 0.035, giving a leak-detection threshold of approximately T = 0.85. Because this threshold is derived from only three intact samples, it should be regarded as a provisional engineering threshold rather than a generalized false-alarm model.

To examine the sensitivity of the detection threshold to the coefficient k, the threshold was recalculated using k = 2, 3, and 4. From the three intact measurements (0.71, 0.78, and 0.75), μ_0_ = 0.7467 and σ_0_ = 0.0351, resulting in thresholds of 0.817, 0.852, and 0.887, respectively. The maximum intact value (0.78) remains below all three thresholds, whereas the minimum leak value (1.17) remains above them. Thus, the present intact and leak records remain separated over this range of k. This analysis evaluates threshold sensitivity within the current dataset and should not be interpreted as validation across independent experimental conditions. The threshold should be re-estimated when pressure difference, sensor coupling, pump operation, background noise, or other acquisition conditions change.

(7) T=0.747+3×0.035≈0.85

A test sample is judged to be in a leak state when

(8) $$\bar{A_{array}}>T$$

otherwise, it is judged as an intact or background-noise state. This threshold is used only for leak/no-leak detection and response-intensity characterization. A robust false-alarm assessment would require additional intact samples, pressure-variation tests, repeated sensor remounting, and background-noise measurements under more realistic operating conditions.

### 3.4. Localization Amplitude Definition and Reference-Sensor Selection

For localization, the feature should describe the spatial distribution of received responses rather than the overall spectral level. Therefore, the RMS amplitude of the i-th channel within the same steady-state window was used as the equivalent received amplitude A_i_:

(9) $$A_{i}=RMS_{i}={\left[\frac{1}{N}\sum_{n}x_{i}{\left[n\right]}^{2}\right]}^{\frac{1}{2}}$$

where N is the number of sampling points in the steady-state window. This localization amplitude is intentionally different from the spectral feature used in the identification stage. The RMS amplitude captures inter-sensor response gradients for ED+MLE localization, whereas the band-averaged spectral amplitude captures the overall frequency-domain separation between intact and leak states.

No additional digital bandpass or high-pass filter was applied before RMS calculation. The RMS amplitude was calculated over the 0.5–1.5 s steady-state interval after mean removal. Frequency-domain processing was performed separately for spectral characterization and leak screening.

To eliminate the unknown leak-source strength and improve relative-amplitude stability, an adaptive reference-sensor strategy was adopted. For each leak record, the RMS amplitudes of the eight channels were calculated, and the channel with the largest RMS amplitude was selected as the reference sensor:

r = arg max_i_ A_i_ (10)

This strategy can be implemented directly from measured data and does not require the true leak-source coordinate. It also avoids using a low-SNR channel as the reference, which would amplify relative-amplitude errors. Nevertheless, it cannot remove all coupling differences among sensors; uncalibrated channel gain remains an important source of localization error.

(11) $$y_{i}=20\log_{10}\left(\frac{A_{i}}{A_{r}}\right)$$

The reference channel itself satisfies y_r = 0 dB. This transformation eliminates the equivalent source strength and reduces sensitivity to absolute amplitude scaling. However, sensor-coupling and channel-gain differences are absorbed into the residual error. Accordingly, the reported localization accuracy should be interpreted in light of uncalibrated channel-gain and coupling differences.

For the representative 1 mm hole leak condition, the channel RMS amplitudes were 0.0557, 0.0687, 0.0599, 0.0680, 0.0574, 0.0705, 0.0593, and 0.0737 V from S1 to S8, respectively. S8 had the largest RMS amplitude and was therefore selected as the reference sensor. The corresponding relative logarithmic amplitudes were −2.43, −0.61, −1.81, −0.70, −2.17, −0.39, −1.89, and 0.00 dB, illustrating how the multi-channel amplitude distribution was converted into the ED+MLE input.

### 3.5. ED+MLE Regional Localization Model

Let the leak source to be localized be located at r = (x, y), and let the position of the i-th sensor be s_i_ = (x_i_, y_i_). The distance from the source to the sensor is

(12) $$d_{i}\left(r\right)=|r-s_{i}{|}_{2}$$

The spatial distribution of multi-channel amplitudes in the plate was modeled as an equivalent energy-decay mapping from a candidate source position to the sensor array [6,8,9,13]. The following amplitude-domain model was adopted:

(13) $$A_{i}=A_{0}g_{i}d_{i}^{-\beta }\exp\left(-\alpha d_{i}\right)+\varepsilon_{i}$$

where A_i_ is the equivalent response amplitude of the i-th sensor, A_0_ is the unknown equivalent source strength, g_i_ is the integrated channel-gain coefficient, α is the equivalent attenuation coefficient, β is the geometric-spreading exponent, and ε_i_ is the noise term. In this study, α and β are not interpreted as pure material constants, because the measured amplitude also includes boundary reflections, Lamb-wave dispersion, multi-modal propagation, and coupling effects. For the geometric spreading exponent, a value of β = 1 was adopted to provide, in combination with the fitted α, an empirically effective decay profile for the present plate geometry and sensor array; this choice compensates, in a lumped manner, for the slower amplitude decay and reflection effects that occur in a bounded plate compared to an infinite medium. The attenuation coefficient α was calibrated from the 1 mm central-hole case. Because independent channel-gain calibration was unavailable, g_i_ was not explicitly corrected and was absorbed into the residual term; this is one reason why the result is reported as regional rather than point localization.

(14) $$L_{i}=20\log_{10}\left(A_{i}\right)$$

Using the reference sensor r to eliminate the unknown source strength, the predicted relative amplitude of the i-th channel at a candidate position r can be written as

(15) $$\hat{y_{i}}\left(r;\alpha \right)=20\log_{10}\left\{\frac{d_{i}{\left(r\right)}^{-\beta }\exp\left[-\alpha d_{i}\left(r\right)\right]}{d_{r}{\left(r\right)}^{-\beta }\exp\left[-\alpha d_{r}\left(r\right)\right]}\right\}$$

The measured relative amplitude is y_i_, and the residual is defined as

(16) $$e_{i}\left(r\right)=y_{i}-\hat{y_{i}}\left(r;\alpha \right)$$

For the purpose of deriving a maximum-likelihood cost function, the logarithmic amplitude residuals of different channels were assumed to be approximately independent and zero-mean Gaussian variables:

(17) $$e_{i}∼N\left(0,\sigma^{2}\right)$$

Under equal channel-noise variance, maximizing the likelihood is equivalent to minimizing the residual sum of squares. The ED+MLE localization problem is therefore written as

(18) $$\hat{r}=\arg\underset{r}{\min}\;J\left(r\right),\;J\left(r\right)=\sum_{i\neq r}e_{i}^{2}\left(r\right)$$

If channel noise variances are obtained in future calibration experiments, a weighted form J(r) = Σ(e_i_^2^/σ_i_^2^) should be used. Because independent channel noise and gain calibration were not available in the present experiment, equal weights were used. Accordingly, the method should be interpreted as an MLE-motivated least-squares ED fitting approach. The localization error is defined as the Euclidean distance between the estimated position and the true defect coordinate:

(19) $$e_{loc}={\left[{\left(\hat{x}-x\right)}^{2}+{\left(\hat{y}-y\right)}^{2}\right]}^{\frac{1}{2}}$$

### 3.6. Equivalent-Attenuation Calibration and Two-Dimensional Search Strategy

Here, α is treated as an equivalent attenuation coefficient rather than a material-specific constant. It represents the lumped effects of material damping, geometric spreading, boundary reflections, guided-wave propagation, and sensor/coupling conditions within the present experimental configuration. In the current implementation, α = 2.3 m^−1^ was empirically selected using the 1 mm central-hole calibration case and was then fixed for the remaining conditions. This value is configuration-dependent and should not be transferred directly to other structures without recalibration.

The 1 mm central-hole leak condition was selected to calibrate α because it had a relatively high signal-to-noise ratio, a known central position, and a concentrated cost-function minimum. The value α = 2.3 m^−1^ was selected based on this calibration case and then fixed for all other hole- and slit-type samples. This calibration strategy improves consistency across conditions, but also introduces a validation limitation: the 1 mm central-hole result should be interpreted strictly as a calibration-case demonstration, whereas the smaller, eccentric, and slit-type defects provide a more meaningful, independent test of transferability.

(20) $$\alpha^{*}=\arg\underset{\alpha }{\min}\;e_{loc}\left(\alpha \right)$$

During localization, candidate leak-source positions were restricted to the aluminum plate region

(21) 0≤x≤500 mm, 0≤y≤500 mm

A two-dimensional grid search was then performed with grid spacing Δx = Δy = 10 mm. For each candidate point, the Euclidean distances d_i_ from the point to the eight sensors were calculated. To avoid singularities when a candidate point coincided with a sensor position, the minimum distance threshold was set to d_min = 1 mm:

(22) $$d_{i}=\max\left(d_{i},d_{min}\right)$$

The candidate point with the minimum ED+MLE cost J(r) was selected as the estimated leak-source position. Because the grid spacing was 10 mm and the model uses an equivalent amplitude decay rather than a full guided-wave propagation model, the results should be interpreted as regional localization. In the context of this study, “regional localization” implies estimating the source position within an area on the order of several tens of millimeters, consistent with the 10 mm grid resolution, rather than achieving a precise point estimate. Errors close to 10 mm approach the grid-resolution limit and should not be presented as sub-grid point-localization accuracy.

### 3.7. Algorithm Implementation Procedure

The complete implementation consists of eight steps: (1) extract the 0.5–1.5 s segment; (2) remove channel means; (3) compute the Hann-windowed normalized single-sided spectrum; (4) calculate Āarray in the 20–200 kHz band; (5) compare it with T = 0.85; (6) for leak records, calculate channel RMS amplitudes and select the maximum-RMS reference channel; (7) compute relative logarithmic amplitudes; and (8) evaluate the ED+MLE cost over the plate grid and report the minimum-cost region.

## 4. Results

### 4.1. Typical Time-Domain Responses and Channel RMS Amplitudes

**Figure 4:**
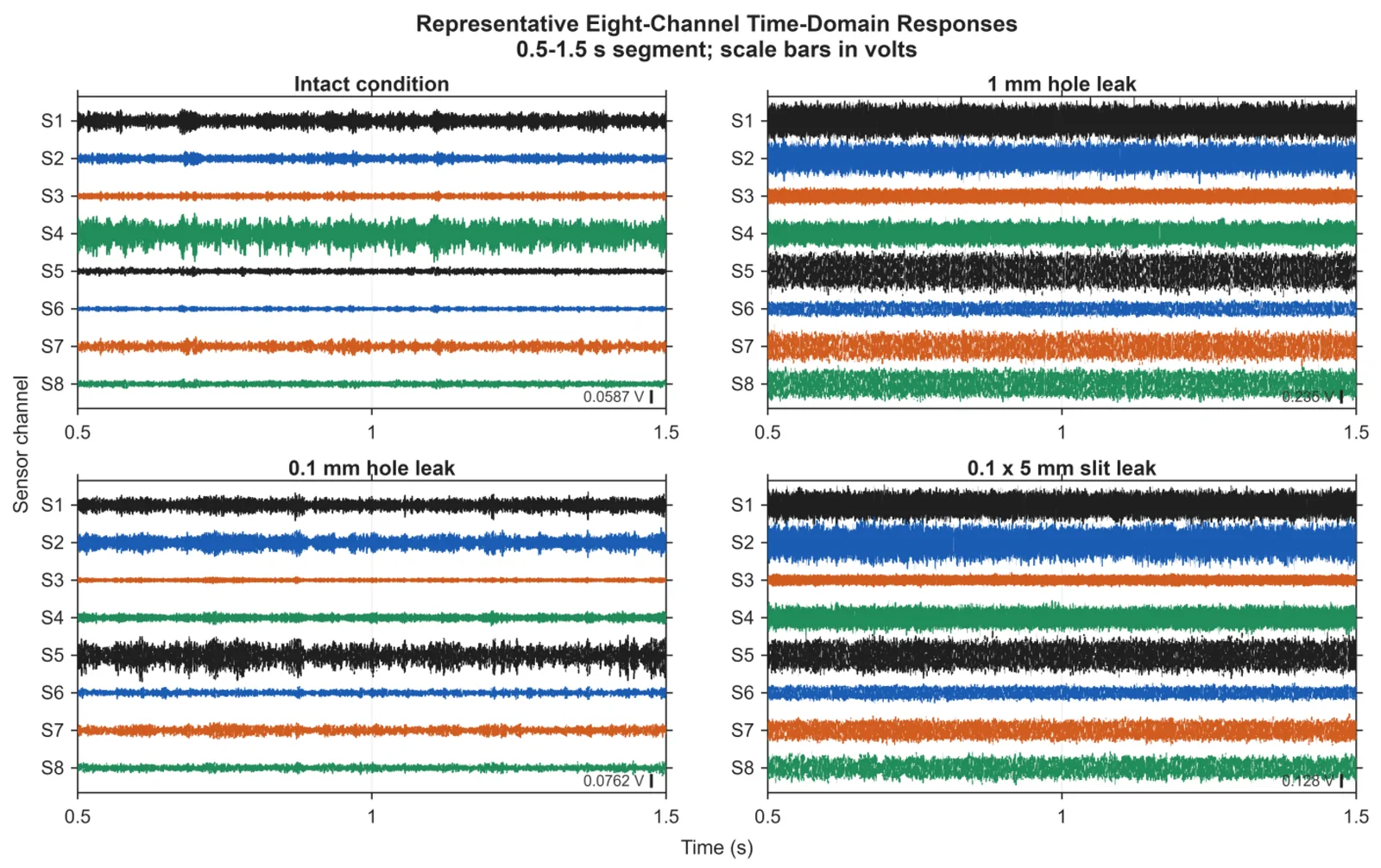
Representative eight-channel time-domain signals. The subplots and axis labels have been enlarged for readability, and channel traces are distinguished by line style and color.

Figure 4 compares representative eight-channel time-domain responses for the intact condition, the 1 mm hole leak, the 0.1 mm hole leak, and the 0.1 mm × 5 mm slit leak. Under continuous leak excitation, the records appeared as sustained random fluctuations rather than isolated burst events with clear first arrivals. Compared with the intact condition, leak cases showed higher fluctuation levels, and different leak intensities produced distinct amplitude distributions across the array. These observations support the methodological choice of using frequency-domain leak detection and amplitude-domain ED+MLE localization instead of relying on first-arrival picking.

The channel RMS amplitudes under the 1 mm hole condition showed clear differences among sensors. These differences contain useful distance- and path-dependent information for localization, but they are also influenced by boundary reflections and channel-coupling variations. For this reason, the localization result is reported as a regional estimate, and the lack of independent channel calibration is treated as a primary uncertainty source.

### 4.2. Spectral Features Under Different Leak States

**Figure 5:**
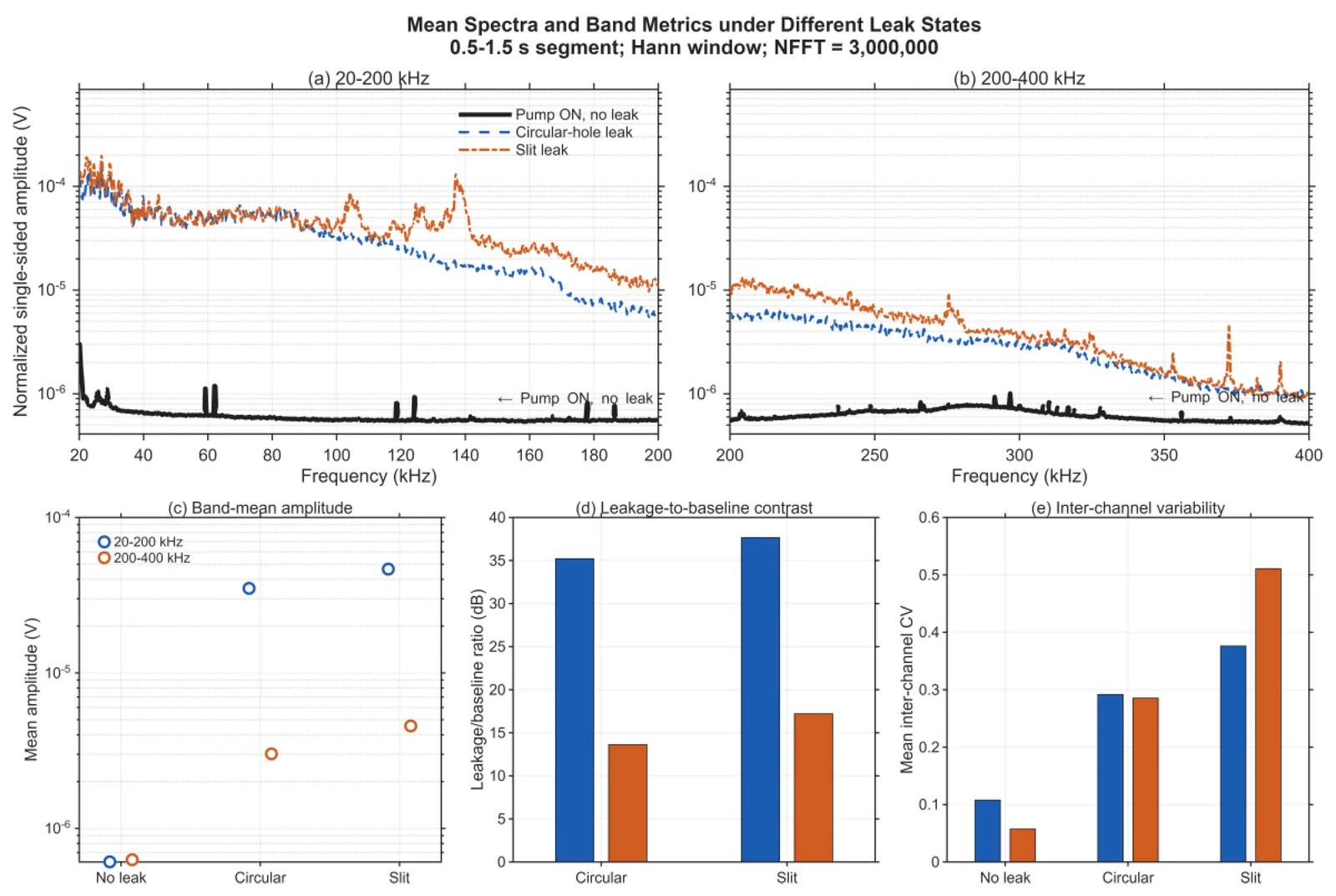
Mean spectra and band metrics under the intact baseline condition (vacuum pump ON, no leak) and representative leak conditions: (**a**) normalized single-sided spectra from 20 to 200 kHz; (**b**) spectra from 200 to 400 kHz; (**c**) band-mean amplitudes; (**d**) leakage-to-baseline ratios; and (**e**) mean inter-channel coefficients of variation. The intact baseline is shown as a thick black line.

Figure 5 compares frequency spectra measured under the intact baseline condition (vacuum pump ON, no leak) and representative leak conditions. The intact baseline is shown as a thick black line. Panels (a) and (b) present the normalized single-sided spectra in the 20–200 kHz and 200–400 kHz ranges, while panels (c)–(e) compare band-mean amplitude, leakage-to-baseline ratio, and inter-channel CV. The leakage-to-baseline contrast decreases by 20.5–21.6 dB in the higher band. The CV response is condition-dependent: it remains similar for circular-hole leaks but increases markedly for the slit-leak condition. Therefore, the 20–200 kHz band provides substantially stronger leak-to-baseline contrast, whereas the 200–400 kHz band offers no consistent inter-channel-variability advantage.

**Figure 6:**
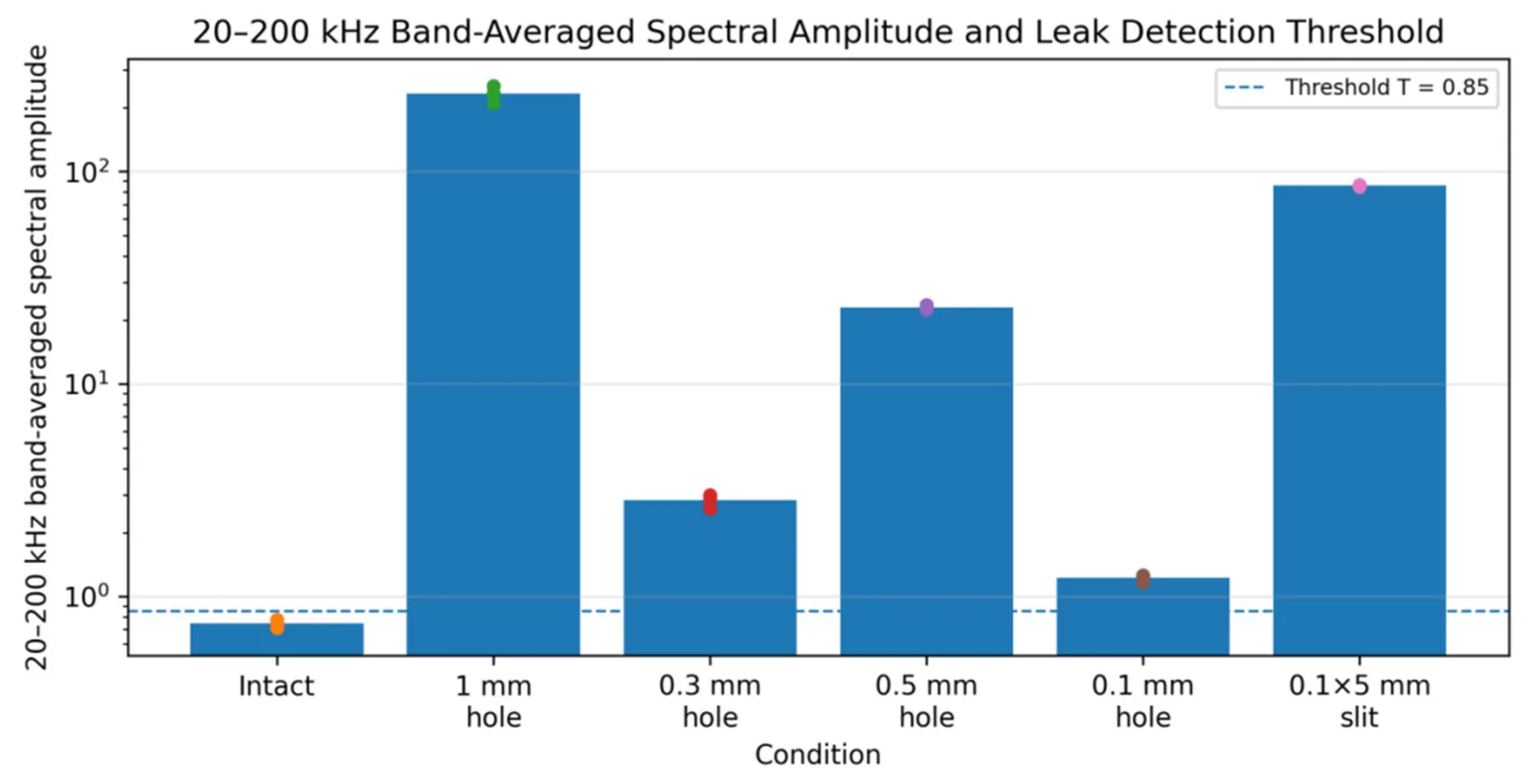
Array-level mean spectral amplitude in the 20–200 kHz band and leak-detection threshold. The colored points denote the three individual repeated measurements (*n* = 3), the bars show the mean values, and the dashed line indicates the detection threshold. The spectral-amplitude values in Figure 6 including the threshold T = 0.85, are expressed in µV.

The intact state is characterized by weak spectral peaks around 59–62 kHz. Hole-leak spectra show enhanced responses mainly around 22–27 kHz. The aperture-dependent amplitude differences among individual hole cases are further quantified in Figure 6 and Table 1. The spectra in Figure 5 are coherent-gain-corrected, normalized single-sided amplitude spectra; therefore, their amplitudes are reported in volts as processed sensor-output amplitudes and may appear in scientific notation. They should not be interpreted as calibrated absolute acoustic-source strengths. The slit leak exhibits a more pronounced broadband response, likely because the opening geometry and effective leak boundary jointly affect the excitation. These trends provide useful engineering interpretation, but they do not by themselves establish a reliable hole/slit classifier.

### 4.3. Mean Spectral Amplitude Statistics and Leak Threshold Discrimination

Figure 6 presents the array-level mean spectral amplitude in the 20–200 kHz band. The dashed line denotes the leak-detection threshold determined from the intact samples, T ≈ 0.85. All three intact samples fall below this threshold, whereas all leak samples in the present dataset exceed it. This demonstrates clear preliminary separability under the current laboratory conditions.

Using T = 0.85, the three intact samples were identified as non-leak samples and the 15 leak samples were identified as leak samples. The observed separation is described as complete separation within the available limited dataset rather than as generalized classification accuracy. Larger independent datasets are required to estimate false-alarm and missed-detection rates.

The mean spectral amplitude in Table 1 generally reflects leak-response intensity under the present geometry, but it is jointly affected by effective opening area, defect type, source position, propagation path, and sensor coupling. The 1 mm hole produces the largest response, whereas the 0.1 mm hole is only slightly above the threshold. Thus, the feature is suitable for leak/no-leak detection and qualitative response-intensity characterization, but it should not be used as the sole feature for defect-geometry classification or leak-rate quantification.

### 4.4. Calibration-Case ED+MLE Demonstration

**Figure 7:**
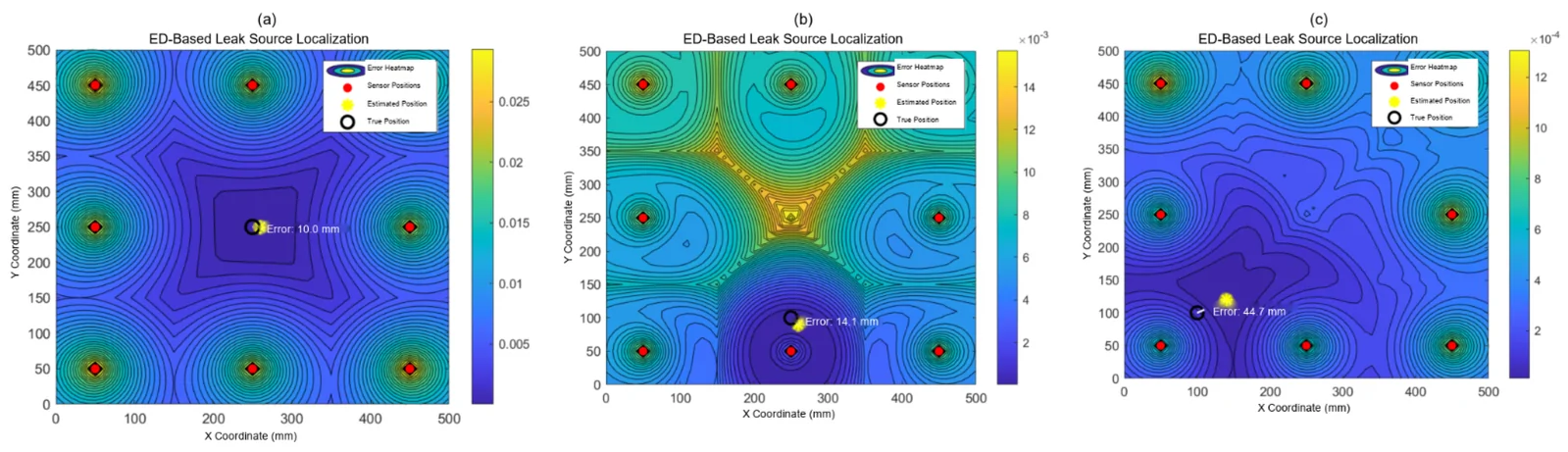
ED+MLE localization heatmaps for representative defect conditions: (**a**) 1 mm central-hole calibration case at (250, 250) mm; (**b**) 0.1 mm × 5 mm slit case at (250, 100) mm; and (**c**) 0.1 mm micro-hole case at (100, 100) mm. The 1 mm central-hole case was used for equivalent-attenuation calibration, whereas the slit and micro-hole cases were used to evaluate transferability under weaker or geometrically different leak conditions. The heatmaps show representative repetitions; statistical localization-error summaries are given in Table 2.

After leak presence was confirmed, the ED+MLE method was used to estimate the leak-source region. Figure 7a shows the ED+MLE cost-function heatmap for the 1 mm central-hole calibration case. The true coordinate was (250, 250) mm, and the estimated coordinate was (250, 260) mm in all three tests, corresponding to a 10 mm grid-level error. Because this condition was also used to select the equivalent attenuation parameter, the result is interpreted strictly as a calibration-case demonstration. It shows that the empirically selected α yielded a well-defined minimum near the strong central leak under the present configuration; it does not establish global parameter optimality or transferability. The remaining cases provide the preliminary transfer assessment under weaker, eccentric, or geometrically different leak conditions.

For the representative 1 mm hole case, the estimated coordinate remained stable across three repeated tests. The concentrated minimum in Figure 7a indicates that strong leak excitation and central placement inside the sensor enclosure provide favorable conditions for ED+MLE localization.

### 4.5. Multi-Condition Localization Heatmap Analysis

Figure 7 compares representative localization cost fields under one calibration condition and two transfer conditions. The 1 mm central hole in Figure 7a produces the most concentrated minimum region, which is consistent with its stronger leak response and central position inside the sensor enclosure. The 0.1 mm × 5 mm slit in Figure 7b still forms a minimum near the true location, but the cost field becomes more asymmetric, reflecting the influence of defect geometry, boundary effects, and array layout. The 0.1 mm micro-hole in Figure 7c yields a flatter and less stable cost field because its weaker response reduces the inter-channel amplitude gradient. These observations explain why localization becomes less accurate when the leak response is weak or when the source lies in a less favorable geometric region.

### 4.6. Localization Error Statistics for Different Apertures and Positions

Table 2 summarizes the localization-error statistics from three tests for each defect. The 1 mm central-hole calibration case yielded a 10 mm error in all tests. The mean errors for the 0.3 mm and 0.5 mm holes were 30.9 mm and 28.9 mm, respectively. The 0.1 mm × 5 mm slit yielded a mean error of 19.6 mm, whereas the 0.1 mm micro-hole yielded the largest mean error, 45.7 mm. These results show that localization accuracy is governed not only by aperture but also by source position, effective opening geometry, signal-to-noise ratio, and the strength of the amplitude gradient across the array.

**Figure 8:**
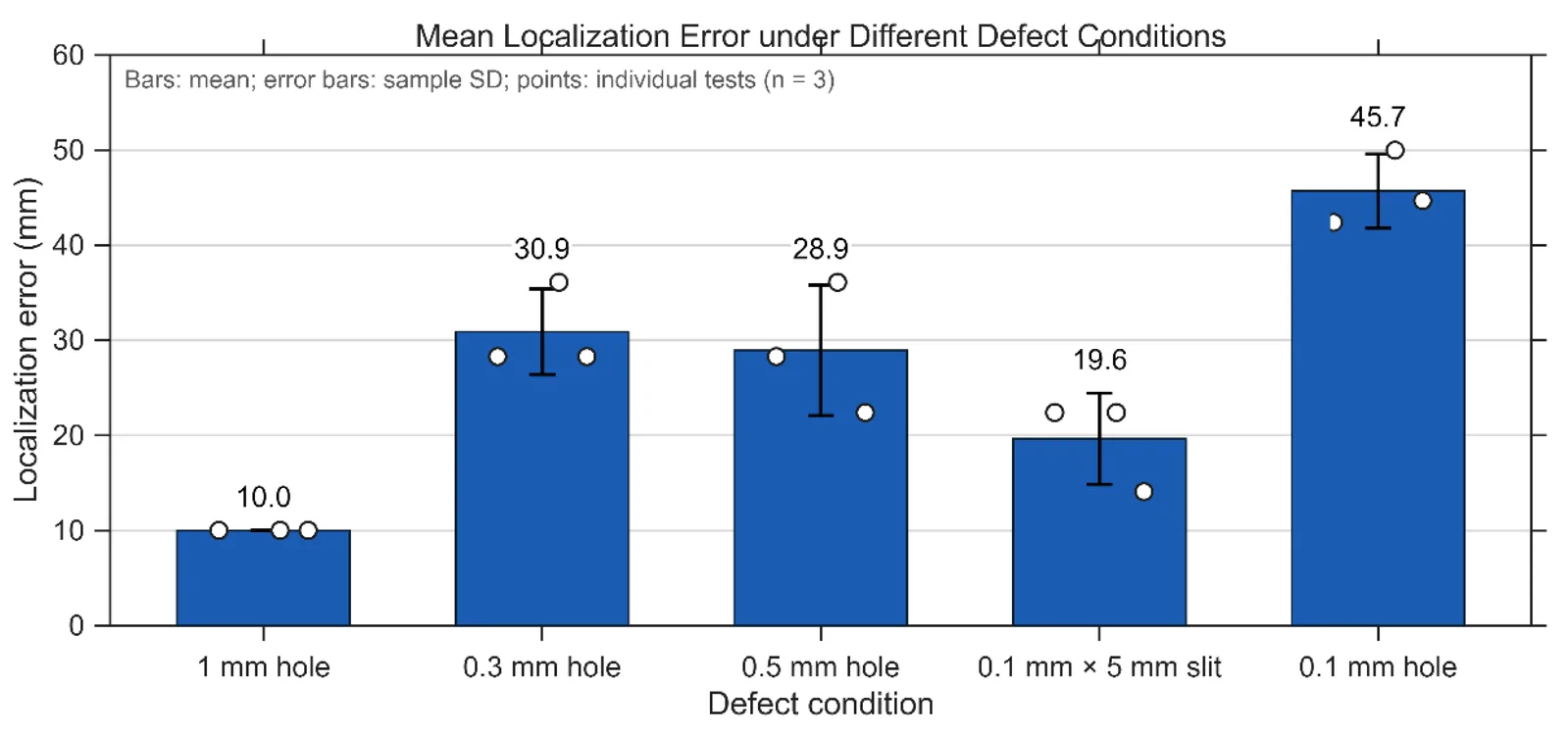
Mean localization error under different defect conditions. Error bars denote the sample standard deviation from three repeated tests (*n* = 3).

Figure 8 should be interpreted descriptively rather than as a single-variable aperture analysis. Central defects benefit from more balanced array geometry and stronger spatial amplitude gradients, whereas eccentric or weak defects produce broader and less stable cost minima, increasing regional localization error. The slit case also demonstrates that a narrow width does not necessarily imply the weakest localization performance; the effective leak boundary length and excitation strength can partly compensate for the small slit width.

## 5. Discussion

For the present continuous leak-AE problem, different localization strategies involve different tradeoffs. TDOA-based methods are highly effective for impulsive AE events with clearly identifiable arrivals, but continuous leak signals in thin plates can make onset picking unreliable because of guided-wave dispersion, multi-modal propagation, and boundary reflections [8,9,14,16,17,18,19]. Beamforming and other array-processing approaches can exploit spatial information from multiple channels, but their performance generally depends on array characterization and additional signal processing [3,14,23]. Amplitude- or energy-decay methods, including the present ED+MLE formulation, avoid explicit onset picking and are computationally light, but they are sensitive to channel gain, coupling variability, and the assumed equivalent attenuation model [7,20,21]. Data-driven approaches can capture complex signal-to-location mappings but usually require substantially larger and more diverse training datasets [12,26]. Accordingly, ED+MLE is presented here as a problem-specific, low-complexity approach for continuous leak AE in spacecraft structural-health-monitoring applications rather than as a universally superior method.

The contribution of this work is the integration of a band-limited screening feature with relative-amplitude regional localization for continuous leak AE signals. In the controlled experiment, the resulting workflow clearly separated intact and leaking conditions and produced mean localization errors of 10.0–45.7 mm across the tested defects while retaining low computational complexity and interpretability.

A deeper analysis of the error characteristics reveals that localization errors do not increase monotonically with decreasing aperture alone. For instance, the 0.1 mm × 5 mm slit, despite its small width, yielded a lower mean error (19.6 mm) than the 0.5 mm eccentric hole (28.9 mm). This can be qualitatively attributed to the slit’s larger effective source dimension and its different excitation spectrum and directivity, which may generate a more favorable amplitude gradient for the given sensor array. Conversely, the 0.1 mm micro-hole’s poor performance (45.7 mm mean error) likely originates from a combination of low SNR, dominance of boundary reflections over the direct amplitude-decay trend, and unbalanced channel sensitivities. These error contributions cannot be quantitatively separated using the present dataset.

Several issues should be addressed in future work to advance this feasibility demonstration toward a more representative monitoring implementation. First, independent channel calibration should be included because the ED+MLE formulation relies on relative channel amplitudes. Repeated Hsu–Nielsen pencil-lead breaks or another reference AE source can be applied under controlled coupling conditions to estimate a relative sensitivity factor g_i_ for each channel [27]. The measured amplitude can then be normalized as A_i_/g_i_, or g_i_ can be incorporated explicitly into the localization model. Second, systematic sensitivity analysis of α, the spreading exponent β, and grid spacing is needed to quantify the stability of the ED+MLE cost minimum and guide parameter selection for different structures and sensor configurations. Third, pressure–time histories and mass-flow leak-rate calibrations should be integrated into the protocol. Finally, validation on structures with stiffeners, curvature, joints, and thermal gradients is required.

## 6. Conclusions

This study proposed an interpretable AE-based workflow for leak monitoring in a thin aluminum-alloy plate under atmospheric-pressure-to-vacuum excitation. The method separates two tasks that are often conflated: leak screening is performed using a 20–200 kHz band-averaged spectral feature, whereas regional localization is performed using the inter-sensor distribution of RMS amplitudes in an ED+MLE cost field.

The controlled experiment showed clear separation between intact and leak records in the available dataset. The ED+MLE model produced a stable grid-level estimate for the 1 mm central-hole calibration case, while smaller and more eccentric defects produced larger errors, with the 0.1 mm micro-hole reaching a mean error of 45.7 mm. These results indicate that the method is most reliable when the leak response is strong and the source lies within a favorable sensor-array geometry.

The current evidence supports feasibility rather than generalization; broader validation requires larger intact/background datasets, independent channel calibration, leak-rate and pressure-history measurements, parameter-sensitivity analysis, and testing on more representative structures. Within these boundaries, the proposed workflow offers a low-complexity and physically interpretable alternative to first-arrival-based localization for continuous leak AE monitoring.

## Figures and Tables

**Table 1:** Array-level mean spectral amplitudes in the 20–200 kHz band and discrimination results under different conditions. The colored points denote the three individual repeated measurements (*n* = 3), the bars show the mean values, and the dashed line indicates the detection threshold. The spectral-amplitude values in Table 1 including the threshold T = 0.85, are expressed in µV.

Condition	Sample 1	Sample 2	Sample 3	Mean	Sample SD	Exceeds Threshold 0.85?
Intact	0.71	0.78	0.75	0.747	0.035	No
1 mm hole	209.2	233.9	252.4	231.8	21.7	Yes
0.3 mm hole	2.6	2.9	3.0	2.8	0.21	Yes
0.5 mm hole	22.7	22.4	23.5	22.9	0.57	Yes
0.1 mm hole	1.17	1.26	1.25	1.2	0.05	Yes
0.1 mm × 5 mm slit	85.0	85.8	86.8	85.9	0.90	Yes

**Table 2:** Localization-error statistics under different defect conditions.

Defect No.	Type	True Coordinate (mm)	Error 1 (mm)	Error 2 (mm)	Error 3 (mm)	Mean Error (mm)	Max. Error (mm)	Min. Error (mm)	Sample SD (mm)
1#	1 mm hole	(250, 250)	10	10	10	10	10	10	0.00
2#	0.3 mm hole	(100, 350)	28.3	36.1	28.3	30.9	36.1	28.3	4.50
3#	0.5 mm hole	(400, 250)	28.3	36.1	22.4	28.9	36.1	22.4	6.87
4#	0.1 mm × 5 mm slit	(250, 100)	22.4	22.4	14.1	19.6	22.4	14.1	4.79
5#	0.1 mm hole	(100, 100)	42.4	50.0	44.7	45.7	50.0	42.4	3.90

## Data Availability

The raw AE waveforms, processed spectral features, localization scripts, and plotting data are available from the corresponding author upon reasonable request, subject to institutional and project-related restrictions.
